# Retraction: Constantin et al. Animal Models in Bladder Cancer. *Biomedicines* 2021, *9*, 1762

**DOI:** 10.3390/biomedicines10010110

**Published:** 2022-01-06

**Authors:** Traian Constantin, Mihai Păvălean, Ștefana Bucur, Maria Magdalena Constantin, Alin Codruț Nicolescu, Irina Pacu, Victor Mădan

**Affiliations:** 1 Faculty of Medicine, “Carol Davila” University of Medicine and Pharmacy, 050474 Bucharest, Romania; traianc29@yahoo.com (T.C.); drmagdadinu@yahoo.com (M.M.C.); nicolescualin66@yahoo.com (A.C.N.); irinapacu@hotmail.com (I.P.); victmad@gmail.com (V.M.); 2 Department of Urology, “Prof. Dr. Th. Burghele” Hospital, 050652 Bucharest, Romania; 3 Department of Urology, Emergency University Central Military Hospital, 010825 Bucharest, Romania; 4 IInd Department of Dermatology, Colentina Clinical Hospital, 020125 Bucharest, Romania; 5 Roma Medical Center for Diagnosis and Treatment, 011773 Bucharest, Romania; 6 Department of Gynecology, “Sfântul Pantelimon” Emergency Hospital, 021659 Bucharest, Romania

The journal retracts the article, “Animal Models in Bladder Cancer. *Biomedicines* **2021**, *9*, 1762” [1]. Following publication, concerns were brought to the attention of the publisher regarding a significant overlap with a previously published article Insights from “Animal Models of Bladder Cancer: Recent Advances, Challenges, and Opportunities *Oncotarget* **2017**, *8*, 57766–57781” [2].

Adhering to our complaint procedure, an investigation was conducted, which revealed an unacceptably high level of similarity between this manuscript and the previously mentioned publication. As a result, this article is retracted. 

This retraction was approved by the Editor in Chief of the journal *Biomedicines*.

The authors agreed to this retraction.
